# Helping editors, peer reviewers and authors improve the clarity, completeness and transparency of reporting health research

**DOI:** 10.1186/1741-7015-6-13

**Published:** 2008-06-16

**Authors:** David Moher, Iveta Simera, Kenneth F Schulz, John Hoey, Douglas G Altman

**Affiliations:** 1 Chalmers Research Group, Children's Hospital of Eastern Ontario Research Institute, Ottawa, ON, Canada; 2 Department of Epidemiology and Community Medicine, Faculty of Medicine, University of Ottawa, Ottawa, ON, Canada; 3 Centre for Statistics in Medicine, University of Oxford, Oxford, UK; 4 Family Health International, Durham, NC, USA; 5 Queen's University, Kingston, ON, Canada

## Abstract

Inadequate reporting is problematic for several reasons. If authors do not provide sufficient details concerning the conduct of their study, readers are left with an incomplete picture of what was done. As such, they are not able to judge the merits of the results and interpret them. The EQUATOR Network is a new initiative aimed at improving the clarity and transparency of reporting health research.

## The reporting of medical research is not clear and transparent: an unacceptable scandal

Mental health continues to be a major health concern in many parts of the world. It is estimated that 5% to 10% of people are affected by depression [1]. In 2006 the United States National Institutes of Health spent US$335 million on research into depression. Yet reports of randomized trials evaluating interventions to optimally manage individuals with depression are disturbingly inadequate, likely making their results of limited use to healthcare professionals, other decision makers and patients. Hotopf and colleagues [2] examined reports of 122 randomized controlled trials (RCTs) evaluating medical interventions for managing individuals with depression and found that only one provided any details about the randomization process used. Empirical evidence shows that inadequate reporting of randomization, a core feature of any randomized trial, is associated with biased estimates of the treatment effect of the order of 20% [3]. Such problems are not unique to reports from depression trials. Inadequate reporting is pervasive to almost every area of health research [4-6]. Without complete, clear and transparent reports, readers cannot judge the reliability and usefulness of health research.

In the late 1970s the International Committee of Medical Journal Editors (ICMJE) published reporting guides for authors. These were limited only to formatting issues [7] although later the ICJME developed the 'Uniform Requirements for Manuscripts Submitted to Biomedical Journals' with a much broader scope [8]. Other efforts have also been made [9,10] although few focused on reporting the design, conduct and analysis of health research. In the mid-1990s the CONSORT Statement, a 22-item checklist and flow diagram, provided what might be considered the first reporting guideline focusing on what should be included in scientific reports of RCTs [11]. Evidence shows that use of CONSORT improves the quality of reporting of RCTs [12]. Journals increasingly find the CONSORT reporting guideline useful; more than 300 of them now directly endorse the CONSORT Statement [13].

The last decade has witnessed considerable activity in developing reporting guidelines: more than 80 of them now exist. They cover a broad spectrum; examples include recommendations for specific study designs (for example, diagnostic accuracy studies [14]), types of data (for example, harms assessed in randomized trials [15]) and sharing of data for microarray experiments (see, for example, [16]). Although a core set of steps are required to optimally develop any reporting guideline, a recent survey of 30 developers of reporting guidelines suggests that most guidelines are created idiosyncratically [17]. Contributing factors include lack of a central repository of available guidelines and a paucity of literature to inform developers of how to develop a reporting guideline [18].

To help improve the quality of reporting health research, we established the EQUATOR (*E*nhancing the *QUA*lity and *T*ransparency *O*f health *R*esearch) Network [19] with initial funding from the UK NHS National Knowledge Service and National Institute for Health Research. This new initiative seeks to improve the quality of scientific publications by promoting transparent and accurate reporting. The network is developing resources and training related to the reporting of health research and will assist in the development, dissemination and implementation of robust reporting guidelines. A free newsletter is available electronically from the Network's website [19].

The Network has five major goals. First, for which there has already been some development, is to build a comprehensive web-based resource centre to develop and maintain up-to-date information, tools and other materials related to reporting health research. One of our first priorities is to develop online resources for editors and peer reviewers, including available literature, related to teaching scientific writing and reporting.

Second, is to set up a network of reporting guideline developers and to initiate and maintain mutual collaboration. Developing reporting guidelines is a new research activity and we are keen to act as a 'bridge' to bring together network members and provide them with scientific support for their guideline development. We are also working to fill the gap in the literature on how to optimally develop reporting guidelines.

Third, is to actively promote reporting guidelines and their use by developing online training courses for editors, peer reviews and researchers, and other activities raising awareness of the importance of reporting guidelines. Given the large number and broad scope of reporting guidelines it is possible that funders of health research will see them as an increasingly helpful resource to ensure that researchers they fund use appropriate guidelines to report their research. While this is at the end of the knowledge generation cycle, funders might also see the reporting guides as an important resource for helping to improve the quality of health research design and conduct.

Fourth, is to conduct a regular assessment of how journals implement reporting guidelines. If reporting guidelines are to achieve their intended goal it is important that they are endorsed appropriately in journals. Recent data indicate substantial room for improvement in this area [20].

Last, is to conduct an annual audit of reporting quality across the health literature. Most journals do not have an objective mechanism for gauging the quality of their published health research. We are proposing to complete an annual audit of the indexed literature and provide this feedback to the journals included on our sample. Continual annual monitoring of the literature will provide unique data on the influence reporting guidelines have on published literature and enable the EQUATOR Network to indirectly monitor its own progress.

The EQUATOR Network will hold its official launch meeting on 26 June 2008 in London, UK. The meeting will focus on a better understanding of the problems associated with health research reporting, the use of reporting guidelines and on finding potential solutions that could lead to an improvement of the health research literature. This is an open meeting that will be preceded by a half-day workshop for editors (see [19]).

In working to achieve these goals the EQUATOR Network will help ensure that the clarity and transparency of healthcare research is optimal for decisions made by healthcare professionals, funders, editors, peer reviewers, and readers.

## Authors' contributions

DM, IS, KFS, JH, and DGA all conceived the paper. DM drafted the initial text and all authors helped edit it. All authors read and approved the final manuscript.

## Pre-publication history

The pre-publication history for this paper can be accessed here:


